# Programmed Cell Death Protein 1–PDL1 Interaction Prevents Heart Damage in Chronic *Trypanosoma cruzi* Infection

**DOI:** 10.3389/fimmu.2018.00997

**Published:** 2018-05-07

**Authors:** Raíssa Fonseca, Rafael Moysés Salgado, Henrique Borges da Silva, Rogério Silva do Nascimento, Maria Regina D’Império-Lima, José Maria Alvarez

**Affiliations:** ^1^Laboratory of Immunology of Infectious Diseases, Department of Immunology, Institute of Biomedical Sciences, University of São Paulo, São Paulo, Brazil; ^2^Center for Immunology, Department of Laboratory Medicine and Pathology, University of Minnesota, Minneapolis, MN, United States

**Keywords:** programmed cell death protein 1, PDL1, regulation, immunosuppression, Chagas disease, chronic Chagas cardiomyopathy, *Trypanosoma cruzi*

## Abstract

Chagas disease is a neglected parasitic infection that affects around six to seven million people, mainly in Latin America. About 30–35% of infected people present chronic Chagas cardiomyopathy (CCC), which eventually leads to death. This condition is characterized by local parasite persistence and leukocyte infiltration. In a murine model of CCC, we observed that among infiltrating leukocytes, CD4^+^ and CD8^+^ T cells were in higher frequency in the heart of chronically infected mice, although elevated expression of the regulatory molecules programmed cell death protein 1 (PD1) and PDL1 suggested these cells could be inhibited. To investigate if PD1–PDL1 interaction in the heart of chronically infected mice negatively impacts on the local immune response, facilitating parasite persistence, and progression to CCC, we attempted to recover the local immune response by treating chronically infected mice with anti-PD1 and anti-PDL1-blocking antibodies together with irradiated *Trypanosoma cruzi*, which provides immune response boosting. Irradiated parasites promote expression of costimulatory molecules in dendritic cells and provide specific parasite antigen, which should aid T cell reactivation upon checkpoint blockade. Following treatment, there was an increased frequency of heart-infiltrating CD4^+^ and CD8^+^ T cells with an effector memory phenotype, an increased histopathology score and decreased heart rate, supporting our previous hypothesis of local immunosuppression induced by this pathway during CCC. In addition, blood parasitemia was reduced, which was associated with increased *T. cruzi-*specific immunoglobulin G 1 antibodies. However, no difference was observed in cytokine production or *T. cruzi* burden in the hearts of treated mice. Taken together, our results suggest PD1–PDL1 interaction protects the heart from excessive immune response.

## Introduction

Chagas disease is caused by the protozoan parasite *Trypanosoma cruzi*. The infection affects around six to seven million people mainly in Latin America, with increasing numbers in non-endemic areas, such as Europe and North America, due to human migration (1). Chronic infection can manifest either as asymptomatic, the indeterminate form, or can progress as chronic Chagas cardiomyopathy (CCC), which is associated with severe cardiac complications in 30–40% of patients (2).

During infection, pro-inflammatory cytokines such as interleukin 12, interferon gamma (IFNγ), and tumor necrosis factor alpha (TNFα) are essential for parasite killing (3, 4). The Th1 immune response coupled with CD8^+^ T cell-mediated cytotoxicity acts at infection sites, such as in the myocardium (5, 6). However, exacerbated cytokine production and cellular response, mainly associated with the Th1 profile, leads to tissue injury accompanied by organ dysfunction (6, 7). Along with the T cell response, parasite-specific antibodies also play an important role in trypomastigote clearance, although immunoglobulin G 1 (IgG1) serum titers also correlate with myocarditis in dogs and mice (8).

To protect organs from tissue damage, lymphoid cells and structural cells, mainly in heart and lung, can express molecules that dampen the immune response (9). Programmed cell death protein 1 (PD1) interaction with PDL1 and PDL2, for example, inhibits T cell activation, proliferation, and cytokine production (10). PD1 ligands have different patterns of expression. While PDL2 can be expressed on dendritic cells and macrophages, PDL1 is constitutively expressed in myeloid, lymphoid, and structural cells and is upregulated by IFNγ (11, 12). PD1 is inducible upon activation in CD4 T cells, CD8 T cells, NKT cells, B cells, and monocytes (13). Elevated expression of inhibitory molecules can contribute to intracellular and extracellular pathogen evasion from the immune system (14, 15).

Checkpoint blockade can reestablish the immune response by blocking key inhibitory interactions and led to parasite and virus burden decrease in different infectious disease models (16, 17). During acute *T. cruzi* infection, both anti-CTLA-4 and anti-PD1 and/or anti-PDL1 monoclonal antibody treatment can improve cellular immune responses and decrease heart parasite load (18, 19). Furthermore, rescue of T cells may need costimulatory signaling through CD28 to improve recovery of immune response in treatment with PD1–PDL1-blocking antibodies (20).

The role of PD1 and PDL1 at chronic *T. cruzi* infection remains unexplored. We hypothesized that blocking of PD1–PDL1 interaction during chronic Chagas disease would restore the immune response and decrease parasite load in blood and tissues. Here, using a mouse model of chronic *T*. cruzi infection that displays heart pathology, we show that checkpoint blockade treatment coupled with irradiated parasites decreases blood parasitemia along with enhanced IgG1 antibody response. The treatment also led to heart damage due to increased leukocyte infiltration, but did not reduce local parasite load. Therefore, while PD1–PDL1 interaction inhibits T and B cell responses, protecting the heart from immune-mediated damage, this may lead to a reduced ability to clear the parasite locally.

## Materials and Methods

### Mice and Parasites

Six- to eight-week-old C3H/HePAS female mice were bred under specific pathogen-free conditions at the Biomedical Sciences Institute at the University of São Paulo (ICB-USP). *T. cruzi* Sylvio X10/4 trypomastigotes, a DTU Type I parasite, were obtained by infection of LLC-MK2 cells as described before (21).

### Infection, Treatment, and Challenge With Irradiated *T. cruzi*

Mice were infected by intraperitoneal (i.p.) inoculation of 1 × 10^6^ trypomastigotes obtained from culture. Mouse treatment was done with anti-PD1 (RMP1-14, BioXCell, USA) and anti-PDL1 (10F.9G2, BioXCell, USA) or rat IgG. Antibody administration was done i.p. through five injections containing 250 µg of antibody every 72 h. Groups challenged with irradiated *T. cruzi* received 1 × 10^6^ parasites i.v. at the first day of antibody treatment. Irradiation was performed with a uniform source of 60 Cobalt (Gammacell, Canada) and *T. cruzi* parasites received 2 kGy. Irradiated parasites were also resuspended in freezing solution (dimethylsulfoxide 50%, FCS 50%) and maintained in liquid nitrogen.

### Ethics Statement

This study was carried out in strict accordance with the Guide for Care and Use of Laboratory Animals of the Brazilian Society of Science in Laboratory Animals (SBCal). The protocols were approved by the Brazilian Biosafety National Committee and the Committee for Animal Ethics (CEUA) of ICB-USP, São Paulo, Brazil under permit numbers 140/10 and 2/2017.

### Electrocardiogram (ECG)

Mice were anesthetized intraperitoneally with 55 ng/g body weight of Ketamine (Imalgene 1000, Merial Inc., USA) and 0.85 ng/g body weight of Xylazine (Rompun 2%, Bayer, Germany). Traces were collected with a Power Lab 4/35 System with a bio-amplifier at 2 mV/s (ADInstruments, USA). Transducers were placed subcutaneously in derivation DII and traces were recorded for 2 min. Beats per minute (BPM) were analyzed using Lab Chart Pro Software (AD Instruments, USA).

### Bone Marrow-Derived Dendritic Cell (BMDC) Culture

Bone marrow cells were harvested, and red blood cells were lysed with ACK lysis buffer. Cells (1 × 10^6^ cell/ml) were cultured in 24-well plates at 37°C with 5% CO_2_ atmosphere in RPMI complete medium, granulocyte–macrophage colony stimulation factor (20 ng/ml) and interleukin-4 (10 ng/ml) (both from Peprotech, USA). The medium was replaced after 3 days in culture. On day 6, non-adherent cells in culture supernatant and loosely adherent cells were harvested by gentle washing with PBS, pooled, and plated at 10^5^ cells/ml/well in 96-well round-bottom plates. In the next day, cells were infected in a ratio of 3:1 irradiated or non-irradiated *T. cruzi* per cell for 24 h, and then harvested for flow cytometry analysis.

### Heart Histopathology

For histopathology analysis, hearts were fixed and embedded in paraffin. Histological longitudinal sections of 7 µm were hematoxylin–eosin stained. For pathology quantification, in each heart section 15 was the maximum score and 0 the lowest one, calculated by the sum of separated partial scores for the atrium (0–5), ventricle pericarditis/endocarditis (0–5), and ventricle/septum myocarditis (0–5), the final global heart score of a mouse being the mean of the scores of all heart slides of this mouse.

### Subpatent Parasitemia

Subpatent parasitemia was screened by hemoculture in Liver Infusion-Tryptose (LIT) medium. Blood was collected from orbital venous sinus and 5 µl aliquots cultured in 1 ml of LIT medium in quintuplicates at 26–28°C. Cultures were examined weekly for living parasites.

### Heart, Spleen, and Peripheral Blood Mononuclear Cell (PBMC) Isolation

For isolation of infiltrating leukocytes, small pieces of hearts were treated with 100 U/ml type IV Collagenase (Sigma Aldrich, USA), RPMI 1640 with 2 mM MgCl_2_, and 2 mM CaCl_2_ for 45 min at 37°C. Spleen single-cell suspensions were treated with ACK lysis buffer to eliminate RBCs. PBMCs were isolated with 74% Percoll gradient.

### Flow Cytometry Analysis

Splenocytes, PBMC, and heart-infiltrating cells were stained with fluorescence-labeled mAbs against CD3 (145-2C11), CD45 (30F-11), CD4 (RM4-5), CD8β (YTS156.7.7), B220 (HIS24), CD11b (M1/70), CD11c (N418), Ly6C (AL-21), CD44 (IM7), CD127 (SB/199), CD62L (MEL-14), CD69 (H1.2F3), CD103 (M290), PD1 (J43), PDL1 (MIH5), CD40 (3/23), CD80 (3/H5), CD86 (GL1), MHC II (10-3.6), IFNγ (XMG1.2), and TNFα (MP6-XT22) all from BD Biosciences, Biolegend, or Affymetrix eBiosciences (USA). For intracellular staining, we used the Cytofix/Cytoperm kit (BD Biosciences) following the manufacturer’s instructions. Cells were analyzed by flow cytometry (FACSCanto or LSR Fortessa; BD Biosciences) with FlowJo 9.5.3. (Tree Star Inc., USA).

### *In Vitro* Stimulation Assays

For proliferation assays, splenocytes were stained with CellTrace Violet (ThermoFischer, USA), following the manufacturer’s instructions. For intracellular assays, cells were maintained in culture with Brefeldin A (3 µg/ml) and Golgistop (1/2,000, final dilution) (BD Biosciences, USA). Cultures containing 1 × 10^6^ cells were kept in RPMI 1640 supplemented with 10% FCS, 2mM l-glutamine, 100 U/ml penicillin, 100 mg/ml streptomycin, 50 µM 2-mercaptoethanol for 72 h (proliferation assays) or 12 h (intracellular cytokine staining assays) at 37°C and 5% CO_2_ atmosphere in the presence of *T. cruzi* antigen (50 µg/ml), anti-CD3 mAb (10 µg/ml; clone 145-2C11), and anti-CD28 mAb (2 µg/ml; clone 37.51) (BD Biosciences, USA), or media. *T. cruzi* Sylvio X10/4 antigen was produced by pelleting parasites obtained from supernatant of LLC-MK2 cell cultures, followed by 20 freeze–thawing cycles and centrifugation at 3,000 rpm to eliminate small clumps. Antigen was stored at 2.5 mg/ml in −80°C and optimal final concentration for *in vitro* lymphocyte stimulation was determined.

### Quantitative Real-Time Polymerase Chain Reaction

Total RNA isolation from heart, muscle, and spleen was isolated with Trizol (Life Technologies, USA) and the RNeasy mini kit (Qiagen, MD, USA) following the manufacturer’s instructions. Reverse transcription was performed with a High Capacity cDNA Reverse Transcription Kit following the manufacturer’s instructions (Applied Biosystems, USA). The cDNA was amplified with specific primers for *Pd1, Pdl1, Pdl2, Ifng, Tnfa, Il10, Tgfb*, and for 18S rRNA of *T. cruzi* (22) and the Universal PCR Master Mix (Applied Biosystems, USA), using the 7500 Real-Time PCR device (Applied Biosystems, USA). The analysis was performed with 7500 Software v2.0.6 (Applied Biosystems, USA).

### Anti-*T. cruzi* IgM, IgG1, and IgG2 Quantification by ELISA

Anti-*T. cruzi* IgM, IgG1, and IgG2 serum levels were quantified by ELISA as described before (23). Briefly, 96-well flat-bottom microtest plates (Costar, USA) were coated overnight at 4°C with 20 µg/ml of a total *T. cruzi* extract. Antibody concentrations were determined using Ig standards with goat anti-mouse Ig isotype peroxidase-conjugated antibodies (Southern Biotechnology Associated, USA). Absorbance was measured at 650 nm with an Epoch Microplate Spectrophotometer (BioTek, USA).

### Immunofluorescence Microscopy

Hearts were frozen in optimum cutting temperature freezing medium and slides of 7-µm sections were prepared. Slides were stained for CD4 (RM4-5), CD8β (YTS156.7.7), and DAPI (Invitrogen), and images were acquired in a Leica DM5500B 4 color fluorescent system. Enumeration of CD4 and CD8 T cells was done manually in Adobe Photoshop and nuclei count was done using ImageJ64 software as previously described (24).

### Statistical Analysis

The results were analyzed with GraphPad Prism 5.0 (GraphPad Software Inc., USA). Data were subjected to the Kolmogorov–Smirnov test to assess Gaussian distribution. Statistical differences were calculated by using unpaired or paired when adequate two-tailed Student’s *t*-test or one-way ANOVA with Tukey post-test. Survival curves were analyzed with Mantel–Cox test. Significant statistical differences between groups were indicated by *p* < 0.05 (*), <0.01 (**), or <0.001 (***).

## Results

### *T. cruzi* Chronic Infection Leads to Heart Pathology and Increased PD1 and PDL1 Expression in Heart-Infiltrating Leukocytes

**Figure 1:**
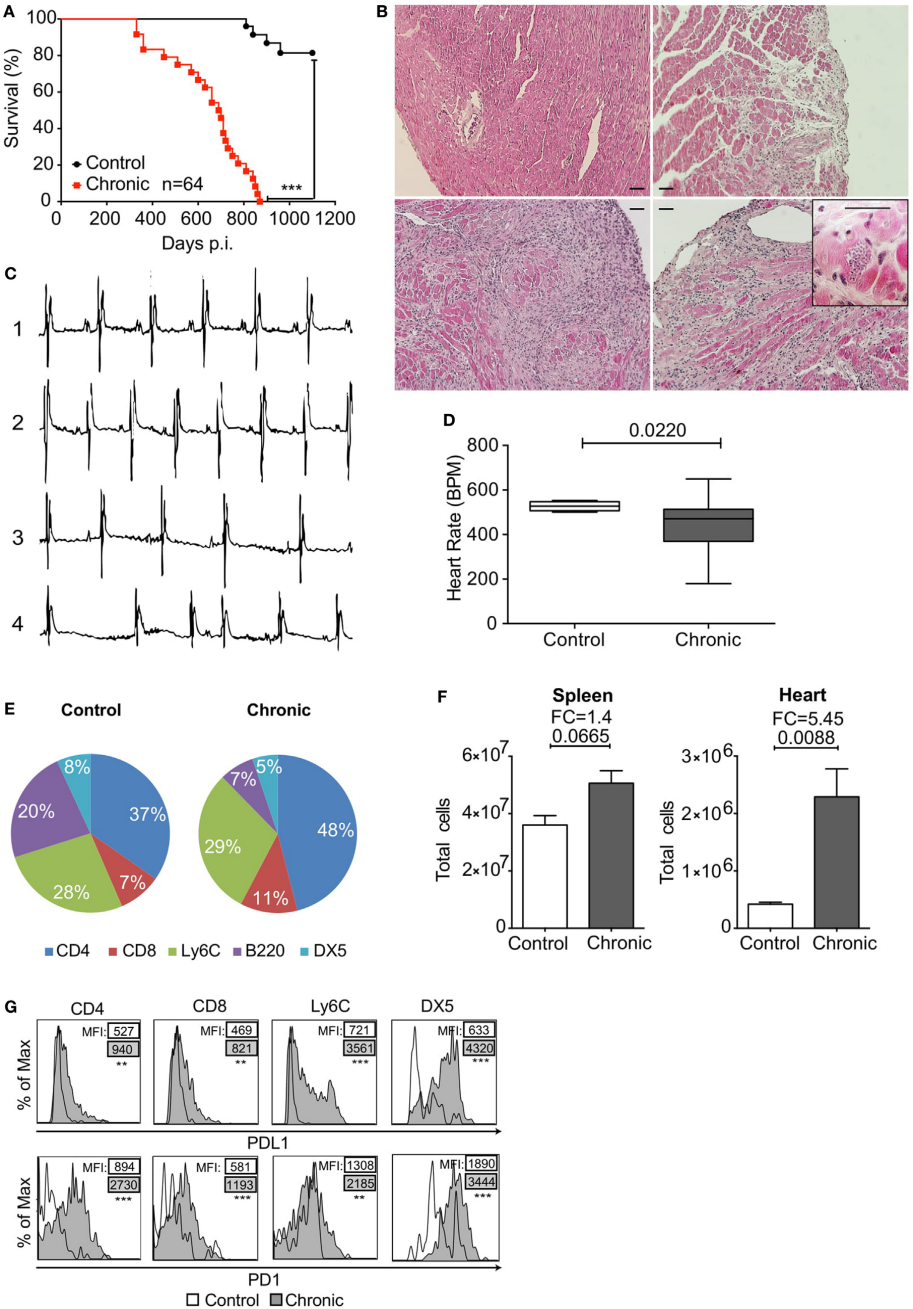
Programmed cell death protein 1 (PD1) and PDL1 expression in heart-infiltrating leukocytes of Sylvio X10/4 *Trypanosoma cruzi*-infected C3H/HePAS mice after 330 days. **(A)** Mortality associated with chronic *T. cruzi* infection. ***p < 0.001, Mantel–Cox test. **(B)** Histological analysis of hearts from an uninfected control mouse (upper left panel) and chronically infected mice with restricted (upper right panel) or extensive (bottom left panel) areas of leukocyte infiltration. Amastigote nest in the heart of a chronically infected mouse (bottom right panel). Bars represent 50 µm. **(C)** Electrocardiogram of healthy non-infected control (1) compared with chronically infected mice with no heart electrical abnormalities (2), bradycardia (3), or arrhythmia (4). **(D)** Heart rate [beats per minute (BPM)] in chronically infected mice in comparison to control mice. Box plots indicate medians (center lines), 25th and 75th percentiles (bottom and top box edges, respectively), minima and maxima (whiskers). *p* Value was assessed with two-tailed Student’s *t*-test. **(E)** Frequencies of CD4^+^ T cells, CD8^+^ T cells, monocytes (Ly6C), B cells (B220), and NK (DX5) cells in the hearts of non-infected and chronically infected mice. All populations were gated in Live^+^/CD45^+^ cells. **(F)** Total cells isolated from spleen and heart of non-infected control and chronically infected mice. **(G)** Histograms showing PDL1 and PD1 expression in each population of heart-infiltrating leukocytes from non-infected (empty line) and chronically infected mice (gray tinted histogram). % of max within each population. Data are representative of three independent experiments (*n* = 5 each).

C3H/HePAS mice infected with *T. cruzi* of the Sylvio X10/4 clone present a chronic phase that displays features of CCC in humans. These mice showed reduced survival in the chronic phase, while no mortality was seen during acute infection (Figure 1A) and exhibited variable degrees of heart inflammatory infiltration at 330 days post infection (dpi) (Figure 1B). The heart pathology ranged from just a few inflammatory cells in small areas, in approximately 30% of infected mice in the chronic phase, to intermediate or extensive areas with leukocyte infiltration and muscle loss, with the presence of rare amastigote nests in 70% of the mice. Moreover, chronically infected mice also presented heart conduction abnormalities, such as arrhythmia (irregular heart beat) and bradycardia (reduced BPM) (Figures 1C,D). In addition, characterization of infiltrating cells showed an increase in CD4^+^ and CD8^+^ T cell population frequency (Figure 1E) and an in total heart-infiltrating cells (Figure 1F). All the identified cell subtypes showed increased PD1 and PDL1 expression compared to the few cells found in the heart of uninfected C3H/HePAS mice (Figure 1G).

### Irradiated *T. cruzi* Increases Dendritic Cell Co-Stimulation and May Be Used as a Tool to Help Recover Inhibited Immune Response

**Figure 2:**
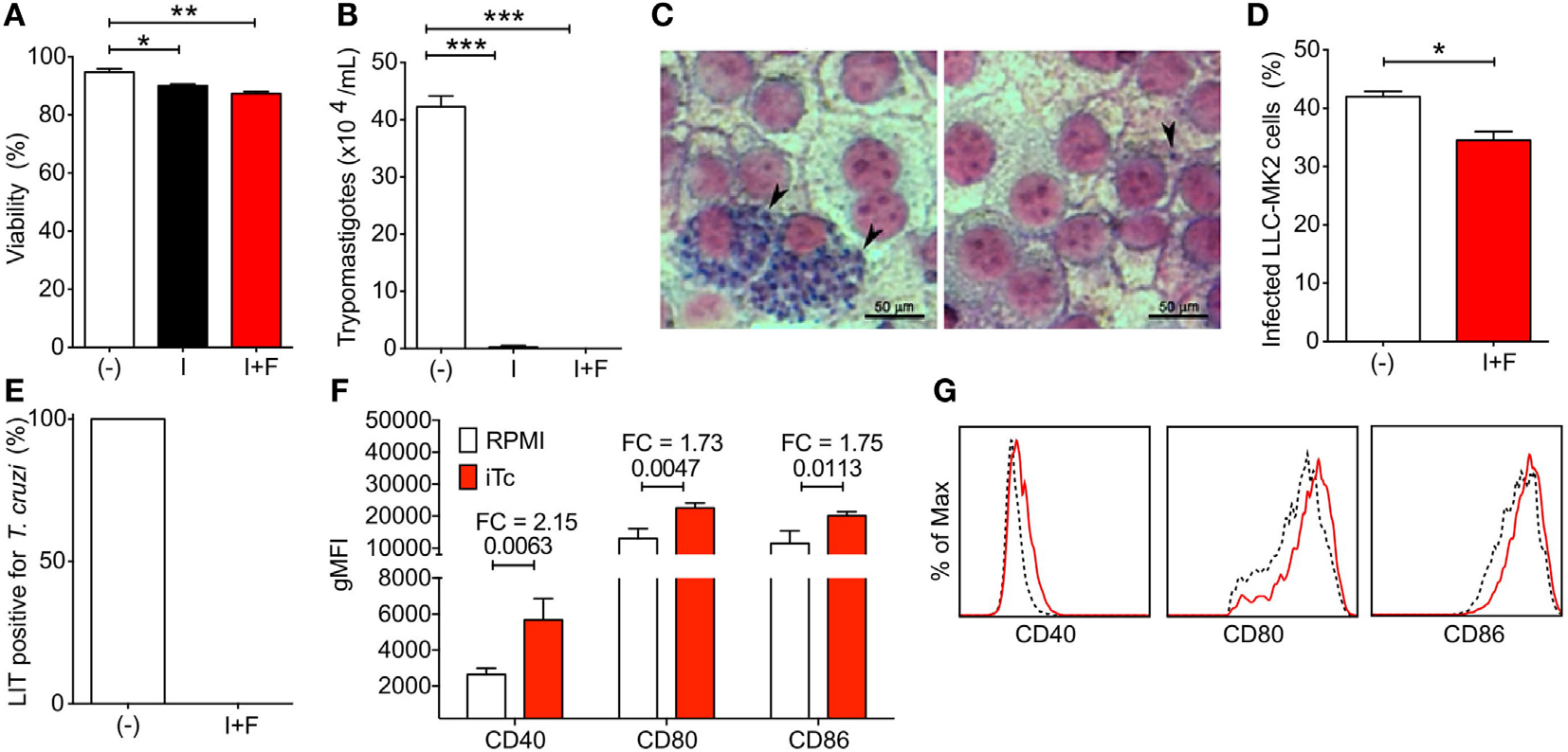
Viability, infection capacity, and bone marrow-derived dendritic cell (BMDC) costimulatory induction of irradiated *Trypanosoma cruzi* parasites. **(A)** Percentage of viability of non-irradiated (−), irradiated (I), and irradiated and frozen (I + F) trypomastigotes. **(B)** Viable trypomastigotes in LLC-MK2 culture supernatant at 4 days post infection (dpi). **(C)** Amastigote nests in LLC-MK2 cells at 4 dpi with non-irradiated (−) and irradiated and frozen (I + F) trypomastigotes. **(D)** Percentage of infected LLC-MK2 cells in cultures described in panel **(C)**. **(E)** Percentage of *T. cruzi*-positive Liver Infusion-Tryptose (LIT) cultures containing 5 µl of blood samples from 7 day-infected mice. **(F)** Geometric mean fluorescence of CD40, CD80, and CD86 expression in non-infected (RPMI) and irradiated *T. cruzi*-infected (iTC) BMDCs. FC, fold change. **(G)** Representative histograms for CD40, CD80, and CD86 expression in non-infected (dashed black line) and irradiated *T. cruzi*-infected (red line) BMDCs. Plots were gated on Live/MHCII^+^CD11c^+^ cells. Statistical differences were evaluated between groups and indicated on graphs with the *p* value of *p < 0.05, **p < 0.01, and ***p < 0.001. Data were expressed as mean ± SD (*n* = 3 each) of one representative experiment out of three.

Intending to restore the immune response, clear the remaining parasites, and resolve the chronic *T. cruzi* infection, we initially treated mice with anti-PDL1 and/or anti-PD1-blocking antibodies. However, no changes in heart pathology or systemic parasitemia were seen following treatment (Figure S1 in Supplementary Material), as well as in T cell populations (data not shown). Because costimulatory signaling has been shown to improve recovery of the immune response following anti-PD1–PDL1 antibody treatment (20), we considered using irradiated parasites to boost the specific immune response by increasing *T. cruzi* antigen availability and the expression of costimulatory molecules. Irradiated parasites (I) presented little viability loss compared with non-irradiated controls (−). No differences were seen in viability of irradiated parasites after freezing (I + F) (Figure 2A). However, trypanosomes were not found in 4-day culture supernatant of irradiated or irradiated and frozen *T. cruzi*-infected LLC-MK2 cells (Figure 2B). Indeed, irradiated and frozen *T. cruzi* maintained infection capacity but did not replicate inside LLC-MK2 cells (Figures 2C,D). Accordingly, when mice were infected with parasites that were frozen and irradiated, viable *T. cruzi* could not be found circulating in blood 7 dpi, which was evaluated through blood culture in LIT media, while all mice infected with non-irradiated parasites were positive (Figure 2E). BMDCs infected with irradiated and frozen *T. cruzi* showed increased expression of CD40, CD80, and CD86 costimulatory molecules (Figures 2F,G). These data corroborate the use of irradiated parasites as a tool to recover the immune response in chronic *T. cruzi* infection by improving antigen presentation.

### PD1 and PDL1 Blockade Associated With Irradiated *T. cruzi* Challenge Increases Heart Pathology and Dysfunction

**Figure 3:**
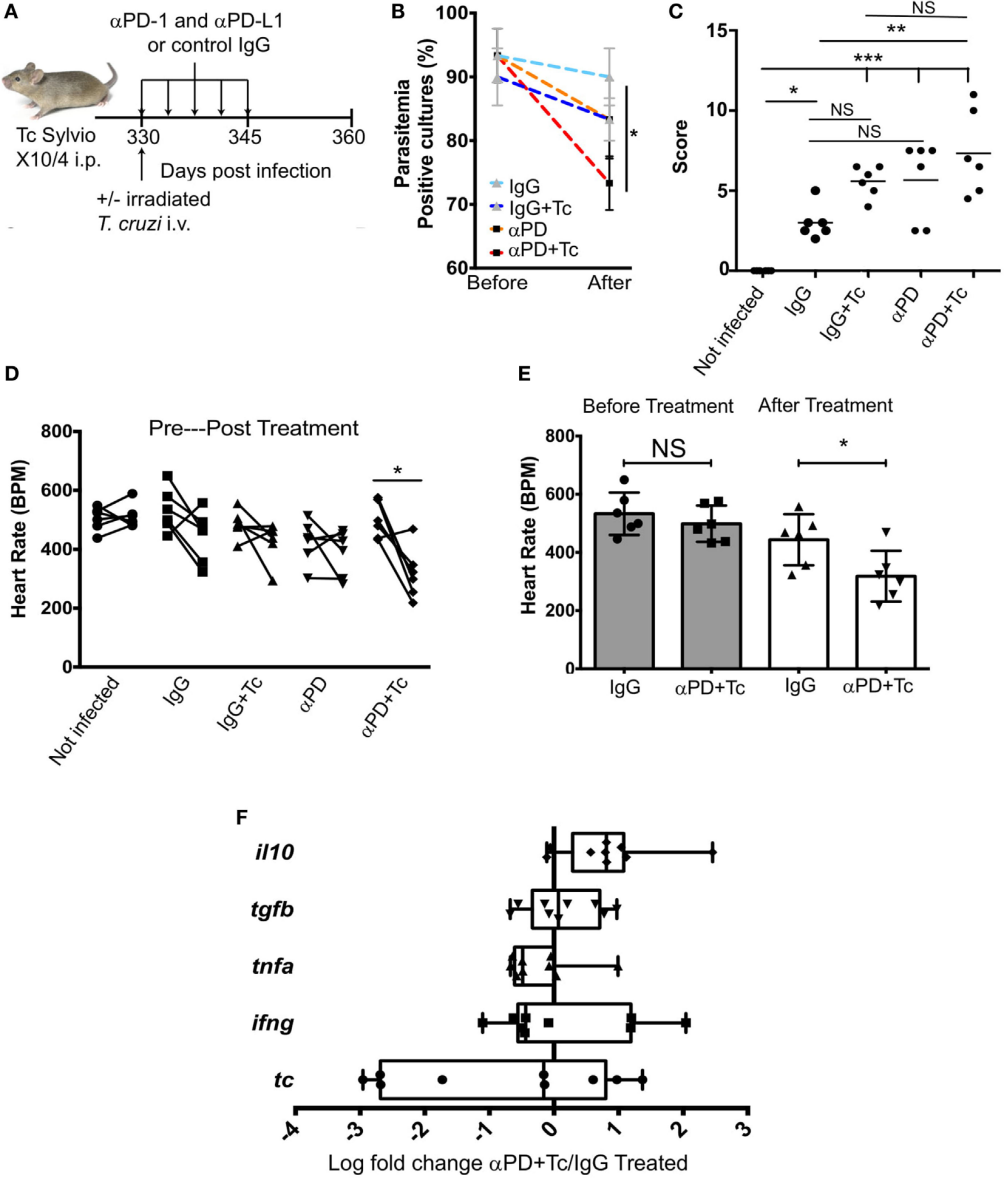
Effects of treating chronically infected mice with anti-programmed cell death protein 1 (PD1), anti-PDL1 associated with irradiated *Trypanosoma cruzi* Sylvio X10/4 challenge. **(A)** Experimental design scheme indicating the treatment. C3H/HePAS mice were infected with 1 × 10^6^ *T. cruzi* parasites. At 330 days post infection, mice were challenged i.v. with 1 × 10^6^ irradiated trypomastigotes and treated with five doses every 3 days (250 µg each) of anti-PD1 and anti-PDL1 blocking antibodies (αPD + Tc). As controls, one mouse group received only control IgG antibody (IgG), a second group received both control IgG and irradiated *T. cruzi* challenge (IgG + Tc), and a third group received anti-PD1 and anti-PDL1-blocking antibodies (αPD). **(B)** Percentage of *T. cruzi*-positive Liver Infusion-Tryptose cultures containing 5 µl of blood samples before and after treatments. **(C)** Histopathology scores attributed to intensity of heart leukocyte infiltration in treated groups. **(D)** Heart rate [beats per minute (BPM)] before and after treatment. **(E)** Heart rate comparison between IgG treated group and αPD + Tc before and after treatment. **(F)** Ratio of αPD + Tc/IgG log fold change expression of *il10, tgfb, tnfa*, and *ifng* genes and *T. cruzi* 18S RNA gene. Samples were normalized to *gapdh*. Statistical differences were evaluated between groups and indicated on graphs with *p < 0.05, **p < 0.01, or ***p < 0.001. Data are grouped from three independent experiments (*n* = 3 each) showing mean ± SD. Box plots indicate medians (center lines), 25th and 75th percentiles (bottom and top box edges, respectively), minima and maxima (whiskers), and individual data points.

To block PD1–PDL1 interaction and provide co-stimulation, chronic mice were injected i.v. with irradiated parasites and i.p. with anti-PDL1 and anti-PD1 blocking antibodies (250 µg each), which was followed by four additional antibody doses every 72 h (αPD + Tc group, Figure 3A). As treatment control, one group received only rat IgG antibody (IgG group). Other two groups were included to assess changes promoted by the irradiated parasites or by checkpoint blockade separately. For that, mice received rat IgG and irradiated *T. cruzi* challenge (IgG + Tc group) or anti-PD1 and anti-PDL1-blocking antibodies (αPD group). Before initiating treatment and after the second dose, blood was collected from IgG and αPD + Tc groups to evaluate early changes in CD4^+^ and CD8^+^ T cell responses. No significant difference in population frequencies was detected at this point (Figure S2 in Supplementary Material).

Heart, spleen, and blood samples were harvested at 15 days post-treatment (360 dpi). The comparison of subpatent parasitemia by blood culture in LIT media before and after treatment revealed a reduction in *T. cruzi*-positive samples obtained from mice of αPD + Tc group compared to IgG group (Figure 3B). Differences between IgG and αPD or IgG + Tc groups were not significant. Histopathological evaluation of leukocyte infiltration in the heart revealed a higher score in αPD + Tc group in comparison to IgG group (Figure 3C). Accordingly, heart BPM in the αPD + Tc group was decreased in comparison to values obtained before treatment (Figure 3D) and the IgG group (Figure 3E). However, we found no differences in *il10, tgfb, tnfa*, and *ifng* gene expression or *T. cruzi* 18S RNA expression in the heart of the αPD + Tc-treated group in comparison to IgG control treatment (log fold change αPD + Tc/IgG treated) (Figure 3F). In summary, PD1 and PDL1 blockade associated with irradiated *T. cruzi* challenge increase heart pathology and dysfunction. However, the cytokine profile of cells and parasite load in the heart are not changed at day 15 post-treatment.

### PD1 and PDL1 Blockade With Irradiated *T. cruzi* Challenge Increases Humoral and Heart Effector Memory T Cell Response

**Figure 4:**
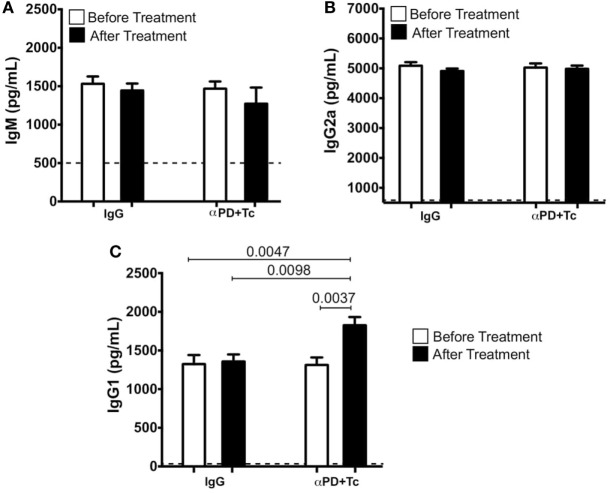
*Trypanosoma cruzi*-specific IgM, IgG2a, and immunoglobulin G 1 (IgG1) serum titers before and after IgG and αPD + Tc treatment. **(A)** IgM, **(B)** IgG2a, and **(C)** IgG1 before [at 330 days post infection (dpi)] and after IgG and αPD + Tc treatment (at 360 dpi). Dashed line represents titers in non-infected control mice serum. Statistical differences were evaluated between groups and indicated on graphs with the respective *p* values. Data were expressed as mean ± SD (*n* = 3 each) of one representative experiment out of four.

*Trypanosoma cruzi-*specific antibody titers were measured in serum before and after IgG and αPD + Tc treatment. Specific IgM (Figure 4A) and IgG2a (Figure 4B) levels were not altered by treatment. However, specific IgG1 titer increased after αPD + Tc treatment, which was also higher in comparison to mice treated with IgG (Figure 4C).

**Figure 5:**
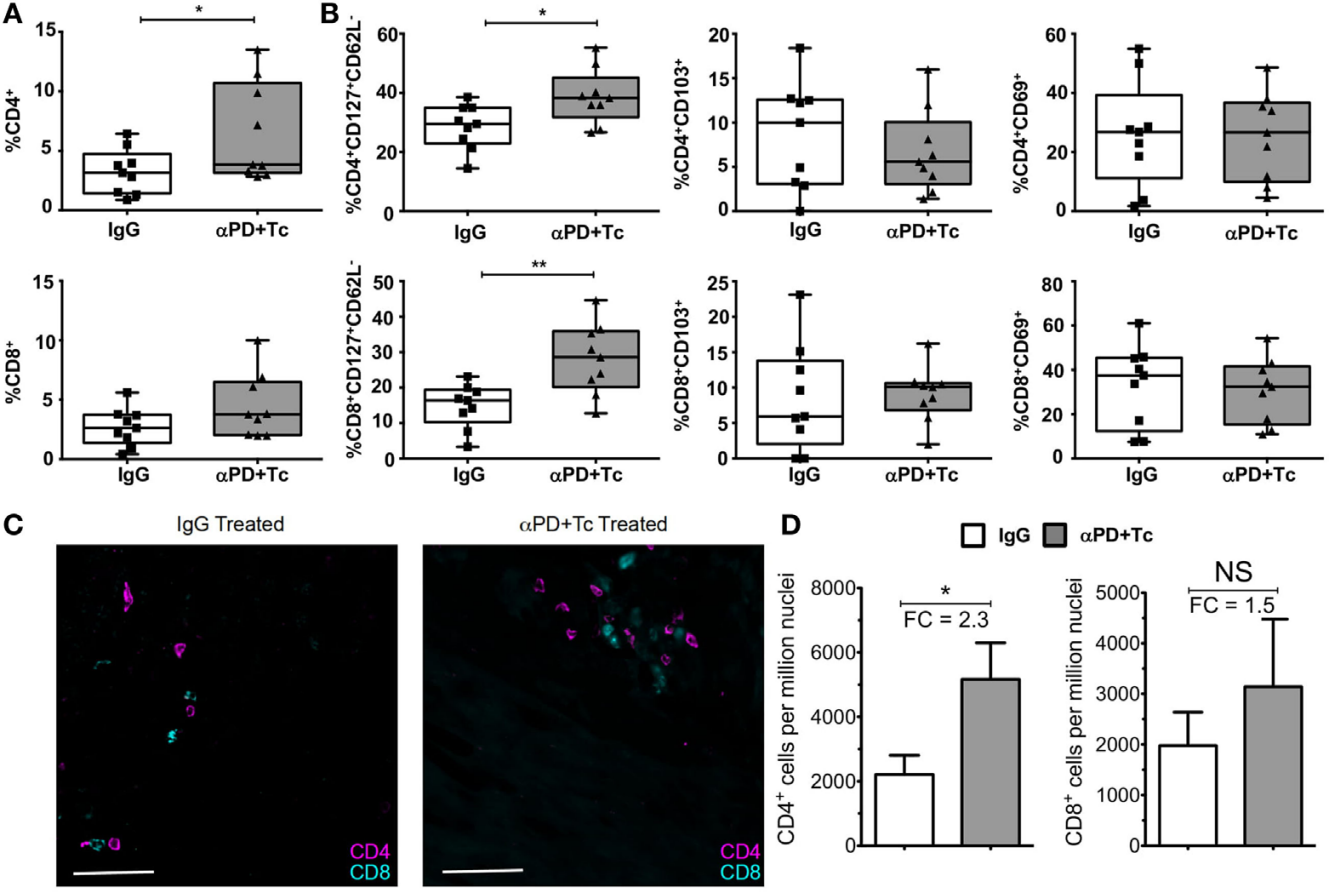
Increase in the percentage of effector memory phenotype in CD4^+^ and CD8^+^ T cells (CD127^+^CD62L^−^) infiltrating the heart of treated mice. **(A)** Percentage of CD4^+^ and CD8^+^ T cells infiltrating the heart of treated chronically infected mice, at day 360 days post infection. Gated in Live^+^ cells. **(B)** Percentage of CD127^+^CD62L^−^, CD103^+^, or CD69^+^ gated in Live^+^CD4^+^ and Live^+^CD8^+^ T cell populations. **(C)** Immunofluorescence staining of CD4^+^ and CD8^+^ T cells in heart sections of treated mice. **(D)** Total CD4^+^ and CD8^+^ T cell frequency relative to DAPI^+^ nucleated cells in heart of treated mice. FC, fold change relative to IgG treated mice. Statistical differences were evaluated between groups and indicated on graphs with *p < 0.05 and **p < 0.01. Data are grouped from three independent experiments (*n* = 3 each). Box plots indicate medians (center lines), 25th and 75th percentiles (bottom and top box edges, respectively), minima and maxima (whiskers), and individual data points (circles).

In addition, we evaluated T cell responses in the heart, spleen, and PBMC after treatment. We observed an increase in CD4^+^ T cell percentage in the heart of αPD + Tc-treated group in comparison to mice that received control IgG, but not in CD8^+^ T cells (Figure 5A). Infiltrating CD4^+^ and CD8^+^ T cells were polarized to an effector memory phenotype (CD127^+^CD62L^−^), but no changes were observed in CD69^+^ or CD103^+^ expression (Figure 5B). We also quantified CD4^+^ and CD8^+^ T cells by immunofluorescence (Figure 5C), and observed an increase of 2.3-fold in CD4^+^ T cells and 1.5-fold in CD8^+^ T cells per million nuclei in αPD + Tc-treated mice in comparison to IgG treated mice (Figure 5D).

However, splenocytes and PBMC from treated chronic mice did not exhibit significant changes in percentages or total numbers of CD4^+^ or CD8^+^ T cells. PBMC did not present changes in CD69 or CD103 expression following treatment (Figure S3 in Supplementary Material). In addition, phenotype, frequencies, and numbers of naïve, central memory, or effector memory T cells were similar between the groups after treatment (Figures S4A,B in Supplementary Material). Also, IFNγ and TNFα production by spleen and blood CD4^+^ and CD8^+^ cells was similar in treated groups following stimulation with anti-CD3 mAb or *T. cruzi* antigen *in vitro* (Figures S5A,B in Supplementary Material). Although all the chronic groups CD4^+^ T cells were able to proliferate in comparison to a control not infected group, none of the treatments could enhance proliferation in CD4^+^ and CD8^+^ T cell post-stimulation *in vitro* (Figures S6A,B in Supplementary Material). Together, these results show that αPD + Tc treatment boosts the anti-*T. cruzi* IgG1 serum antibody production and the T cell response in the heart.

## Discussion

The chronic inflammatory process in the heart of patients with CCC seems to be due to local parasite persistence (25). Yet, because the anti-*T. cruzi* available drugs are not totally efficient in reducing patients’ progression to cardiomyopathy, new therapeutic approaches with better perspectives are needed. Benzonidazole treatment was shown to significantly reduce blood parasite detection by PCR analysis in chronically infected patients, but it was not able to reduce cardiac deterioration (26). At present, it is not known what causes heart parasite persistence in one-third of *T. cruzi*-infected individuals and how this relates to CCC, the most prevalent hypothesis being the inefficiency of the local anti-*T. cruzi* response. To evaluate if heart parasites could be eliminated by increasing the immune response, we used C3H/HePAS mice infected with *T. cruzi* Sylvio X10/4 parasites, an infection model that develops a chronic heart pathology with some similarities to CCC in humans (27). In addition to heart leukocyte infiltration and increased mortality, chronically infected C3H/HePAS mice displayed decreased heart rate, as well as strong variability in the intensity of local pathology and parasite load (data not shown), which are probably related to the extended survival of some mice. Although these mice presented more CD4^+^ and CD8^+^ T cell recruitment in the heart, increased expression of PD1 and PDL1 suggested an inhibition of the heart immune response that could be permissive to parasite perpetuation. The increased expression of inhibitory receptors has been proposed, in addition to *T. cruzi* escape mechanisms, to contribute to parasite persistence, and hence to chronic heart pathology (28).

In acute *T. cruzi* infection, mice treated with anti-PD1, anti-PDL1, or anti-PDL2 antibodies displayed increased cardiac inflammatory response and reduced parasitemia, along with increased mortality rates (18). Checkpoint blockade benefited the outcome of other infectious diseases by increasing CD8^+^ T cell proliferation and cytokine production, leading to diminished viral load (10, 29). Nevertheless, the involvement of PD1–PDL1 regulatory interaction in chronic Chagas disease has remained unstudied. Initially, we used anti-PD1 and anti-PDL1-blocking antibodies to drive immune response restoration, but no change was observed in heart parasite load or pathology and blood parasitemia in comparison to rat IgG-treated mice (unpublished data). Because costimulatory signaling has been shown to improve immune response restoration in anti-PD1 treatment (20), we used irradiated parasites to potentiate immune response restoration in association with checkpoint blockade. These parasites were viable and could infect cells but were not replicative *in vivo* or *in vitro*. Also, irradiate parasites worked to increase expression of costimulatory molecules on dendritic cells.

Subpatent parasitemia decreased in mice that received the anti-PD1 and anti-PD-L1 antibody treatment associated with irradiated parasite challenge (αPD + Tc group), probably as a result of increased anti-*T. cruzi* IgG1 in blood, as previous studies have shown the importance of IgG1 in removing parasites from the circulation (30). Also, a positive correlation in IgG1 titers and levels of heart inflammation have been demonstrated before (8). This correlation supports the increase in the histopathology score found in mice following treatment, ultimately leading to impaired function, verified through a decrease in BPM. The unaltered IgG2a production after treatment is in consonance with unaffected IFNγ production by CD4^+^ and CD8^+^ T cells both in spleen and blood, as IFNγ is known to promote antibody class switch to IgG2a (31). In accordance with our results, previous studies reported that PD1–PDL1 interaction plays a crucial role in regulating the humoral immune response. PDL1^hi^ B cells suppress follicular helper T cells (T_FH_) in the germinal center decreasing antibody production (32). When treated with PD1-blocking antibody, macaque survival was improved in SIV infection by increasing antibody titers (33).

In the heart, a rise in the intensity of inflammatory infiltrate coupled with an increase in the frequency of CD4^+^ and CD8^+^ T cells with an effector memory phenotype were observed in αPD + Tc-treated mice. While we expected to find changes in pro-inflammatory or anti-inflammatory cytokine production due to immune response reactivation or secondary inhibitory mechanisms to avoid tissue damage, no change in *tnfa, ifng il10*, and *tgfb* gene transcription was seen locally 15 days post-treatment. The lack of changes in heart parasite load following αPD + Tc treatment indicate that the increased infiltration of effector memory CD4^+^ and CD8^+^ T cells in this compartment was not enough for local parasite elimination. The parasite persistence could be associated with failure of αPD + Tc treatment to increase IFNγ and TNFα production by infiltrating T cells. It is also possible that *T. cruzi* clearance in the heart requires other elements, such as parasite sensing by cardiomyocytes and consequent production of “find me signals” for effector T cells (27). Lack of leukocytes around amastigote nests indicating that leukocytes are not acting to clear the parasite replication site has been described previously by histopathology studies in the hearts from Chagas disease patients and *T. cruzi*-infected mice (27, 34).

Even though the αPD + Tc treatment failed to reduce parasite load in the heart, it resulted in increased heart damage. This finding suggests that PD1 and PDL1 expression by heart structural cells and immune cells is necessary to prevent tissue damage that results from local immune response. In support of this, high levels of plasma IFNγ, TNFα, (IL-1β), and IL-6 correlated with the severity of dilated cardiomyopathy in chagasic patients (35), indicating that exacerbated immune can cause local damage. Endorsing the role of pro-inflammatory factors in heart damage, the treatment of chronic mice with pentoxifylline, which has anti-inflammatory properties, could reduce plasma levels of TNFα and CD8 T cell response, causing decreased heart inflammation and recovering its function (36). These results demonstrate that CCC is not simply due to a deficient anti-*T. cruzi* immune response.

Heart inflammation seems to have a dualistic role in chronic *T. cruzi* infection. While it aids in parasite control, it can cause tissue damage. Hence, our results revealed the importance of the PD1–PDL1 inhibitory interaction in heart preservation in CCC. In other disease models, heart endothelial expression of PDL1 was crucial to control cardiac injury and leukocyte inflammation, emphasizing that PD1–PDL1 blockade can lead to heart damage (37). In conclusion, our data demonstrate that PD1–PDL1 interaction is important to protect the heart from damage caused by infiltrating leukocytes. Allowing local parasite persistence is the price paid by limiting the immune response to prevent local damage. New experiments associating the immune response boosting described in this study with fibrosis inhibition and myocyte reparation to protect heart function could have promising results. However, being unable to clear local parasite burden, unless a very clear understanding is reached in *T. cruzi* escape mechanisms and new therapeutic means are devised to facilitate parasite clearance, we speculate CCC will not be simply resolved.

## Ethics Statement

This study was carried out in strict accordance with the Guide for Care and Use of Laboratory Animals of the Brazilian Society of Science in Laboratory Animals (SBCal). The protocols were approved by the Brazilian Biosafety National Committee and the Committee for Animal Ethics (CEUA) of ICB-USP, São Paulo, Brazil under permit numbers 140-10 and 2-2017.

## Author Contributions

RF, RS, HB, and RN performed the experiments; RF analyzed data; MD-L and JA contributed critical reagents and experimental help; RF and JA wrote the manuscript; JA was responsible for research supervision, coordination, and strategy.

## Conflict of Interest Statement

The authors declare that the research was conducted in the absence of any commercial or financial relationships that could be construed as a potential conflict of interest.

## References

[1] WHO. Weekly Epidemiological Record. World Health Organization (2015). p. 1–12.

[2] Andrade JP Marin Neto JA Paola AAV Vilas-Boas F Oliveira GMM Bacal F I Latin American Guidelines for the diagnosis and treatment of Chagas’ heart disease: executive summary. Arq Bras Cardiol (2011) 96:434–42. 10.1590/S0066-782X2011000600002 21789345

[3] Reed SG. In vivo administration of recombinant IFN-gamma induces macrophage activation, and prevents acute disease, immune suppression, and death in experimental *Trypanosoma cruzi* infections. J Immunol (1988) 140:4342–7. 3131431

[4] Lima EC Garcia I Vicentelli MH Vassalli P Minoprio P. Evidence for a protective role of tumor necrosis factor in the acute phase of *Trypanosoma cruzi* infection in mice. Infect Immun (1997) 65:457–65. 9009297 10.1128/iai.65.2.457-465.1997 PMC174617

[5] Fonseca SG Reis MM Coelho V Nogueira LG Monteiro SM Mairena EC Locally produced survival cytokines IL-15 and IL-7 may be associated to the predominance of CD8 +T cells at heart lesions of human chronic Chagas disease cardiomyopathy. Scand J Immunol (2007) 66:362–71. 10.1111/j.1365-3083.2007.01987.x 17635814

[6] Nogueira LG Santos RHB Fiorelli AI Mairena EC Benvenuti LA Bocchi EA Myocardial gene expression of T-bet, GATA-3, Ror-γt, FoxP3, and hallmark cytokines in chronic Chagas disease cardiomyopathy: an essentially unopposed TH1-type response. Mediators Inflamm (2014) 2014:914326–9. 10.1155/2014/914326 25152568 PMC4134835

[7] Gomes JAS Bahia-Oliveira LMG Rocha MOC Busek SCU Teixeira MM Silva JS Type 1 chemokine receptor expression in Chagas’ disease correlates with morbidity in cardiac patients. Infect Immun (2005) 73:7960–6. 10.1128/IAI.73.12.7960-7966.2005 16299288 PMC1307097

[8] Caldas IS de Figueiredo Diniz L Guedes PMDM Nascimento ÁFDSD Galvão LMDC de Lima WG Myocarditis in different experimental models infected by *Trypanosoma cruzi* is correlated with the production of IgG1 isotype. Acta Trop (2017) 167:40–9. 10.1016/j.actatropica.2016.12.015 27993495

[9] Freeman GJ Wherry EJ Ahmed R Sharpe AH Reinvigorating exhausted HIV-specific T cells via PD-1-PD-1 ligand blockade. J Exp Med (2006) 203:2223–7. 10.1084/jem.20061800 17000870 PMC2118103

[10] Barber DL Wherry EJ Masopust D Zhu B Allison JP Sharpe AH Restoring function in exhausted CD8 T cells during chronic viral infection. Nature (2005) 439:682–7. 10.1038/nature04444 16382236

[11] Ishida M Iwai Y Tanaka Y Okazaki T Freeman GJ Minato N Differential expression of PD-L1 and PD-L2, ligands for an inhibitory receptor PD-1, in the cells of lymphohematopoietic tissues. Immunol Lett (2002) 84:57–62. 10.1016/S0165-2478(02)00142-6 12161284

[12] Liang SC Latchman YE Buhlmann JE Tomczak MF Horwitz BH Freeman GJ Regulation of PD-1, PD-L1, and PD-L2 expression during normal and autoimmune responses. Eur J Immunol (2003) 33:2706–16. 10.1002/eji.200324228 14515254

[13] Agata Y Kawasaki A Nishimura H Ishida Y Tsubata T Yagita H Expression of the PD-1 antigen on the surface of stimulated mouse T and B lymphocytes. Int Immunol (1996) 8(5):765–72. 8671665 10.1093/intimm/8.5.765

[14] Kirchberger S Majdic O Steinberger P Bluml S Pfistershammer K Zlabinger G Human rhinoviruses inhibit the accessory function of dendritic cells by inducing sialoadhesin and B7-H1 expression. J Immunol (2005) 175:1145–52. 10.4049/jimmunol.175.2.1145 16002716

[15] Smith P Walsh CM Mangan NE Fallon RE Sayers JR McKenzie ANJ *Schistosoma mansoni* worms induce anergy of T cells via selective up-regulation of programmed death ligand 1 on macrophages. J Immunol (2004) 173:1240–8. 10.4049/jimmunol.173.2.1240 15240716

[16] Butler NS Moebius J Pewe LL Traore B Doumbo OK Tygrett LT Therapeutic blockade of PD-L1 and LAG-3 rapidly clears established blood-stage *Plasmodium* infection. Nat Immunol (2011) 13:188–95. 10.1038/ni.2180 22157630 PMC3262959

[17] Lee J Ahn E Kissick HT Ahmed R. Reinvigorating exhausted T cells by blockade of the PD-1 pathway. For Immunopathol Dis Therap (2015) 6:7–17. 10.1615/ForumImmunDisTher.2015014188 28286692 PMC5341794

[18] Gutierrez FRS Mariano FS Oliveira CJF Pavanelli WR Guedes PMM Silva GK Regulation of *Trypanosoma cruzi*-induced myocarditis by programmed death cell receptor 1. Infect Immun (2011) 79:1873–81. 10.1128/IAI.01047-10 21357717 PMC3088162

[19] Martins GA Tadokoro CE Silva RB Silva JS Rizzo LV. CTLA-4 blockage increases resistance to infection with the intracellular protozoan *Trypanosoma cruzi*. J Immunol (2004) 172:4893–901. 10.4049/jimmunol.172.8.4893 15067068

[20] Kamphorst AO Wieland A Nasti T Yang S Zhang R Barber DL Rescue of exhausted CD8 T cells by PD-1-targeted therapies is CD28-dependent. Science (2017) 355:1423–7. 10.1126/science.aaf0683 28280249 PMC5595217

[21] Miles MA Letter: cloning *Trypanosoma cruzi*. Trans R Soc Trop Med Hyg (1974) 68:256 10.1016/0035-9203(74)90126-6 4608041

[22] Sardinha LR Mosca T Elias RM do Nascimento RS Gonçalves LA Bucci DZ The liver plays a major role in clearance and destruction of blood trypomastigotes in *Trypanosoma cruzi* chronically infected mice. PLoS Negl Trop Dis (2010) 4:e578. 10.1371/journal.pntd.0000578 20052269 PMC2793026

[23] Marinho CRF Bucci DZ Dagli MLZ Bastos KRB Grisotto MG Sardinha LR Pathology affects different organs in two mouse strains chronically infected by a *Trypanosoma cruzi* clone: a model for genetic studies of Chagas’ disease. Infect Immun (2004) 72:2350–7. 10.1128/IAI.72.4.2350-2357.2004 15039360 PMC375186

[24] Steinert EM Schenkel JM Fraser KA Beura LK Manlove LS Igyártó BZ Quantifying memory CD8 T cells reveals regionalization of immunosurveillance. Cell (2015) 161:737–49. 10.1016/j.cell.2015.03.031 25957682 PMC4426972

[25] Monteón-Padilla V Hernández-Becerril N Ballinas-Verdugo MA Aranda-Fraustro A Reyes PA. Persistence of *Trypanosoma cruzi* in chronic chagasic cardiopathy patients. Arch Med Res (2001) 32:39–43. 10.1016/S0188-4409(00)00261-7 11282179

[26] Morillo CA Marin Neto JA Avezum A Sosa-Estani S Rassi A Rosas F Randomized trial of benznidazole for chronic Chagas’ cardiomyopathy. N Engl J Med (2015) 373:1295–306. 10.1056/NEJMoa1507574 26323937

[27] Marinho CRF Nuñez-Apaza LN Bortoluci KR Bombeiro AL Bucci DZ Grisotto MG Infection by the Sylvio X10/4 clone of *Trypanosoma cruzi*: relevance of a low-virulence model of Chagas’ disease. Microbes Infect (2009) 11:1037–45. 10.1016/j.micinf.2009.07.011 19660570

[28] Argüello RJ Albareda MC Alvarez MG Bertocchi G Armenti AH Vigliano C Inhibitory receptors are expressed by *Trypanosoma cruzi*-specific effector T cells and in hearts of subjects with chronic Chagas disease. PLoS One (2012) 7:e35966–35913. 10.1371/journal.pone.0035966 22574131 PMC3344843

[29] Fuller MJ Callendret B Zhu B Freeman GJ Hasselschuwert DL Satterfield W Immunotherapy of chronic hepatitis C virus infection with antibodies against programmed cell death-1 (PD-1). Proc Natl Acad Sci U S A (2013) 110:15001–6. 10.1073/pnas.1312772110 23980172 PMC3773803

[30] Brodskyn CI Silva AM Takehara HA Mota I. IgG subclasses responsible for immune clearance in mice infected with *Trypanosoma cruzi*. Immunol Cell Biol (1989) 67(Pt 6):343–8. 10.1038/icb.1989.50 2516504

[31] Nguyen HV Mouly E Chemin K Luinaud R Despres R Fernand J The Ets-1 transcription factor is required for Stat1-mediated T-bet expression and IgG2a class switching in mouse B cells. Blood (2012) 199:4174–81. 10.1182/blood-2011-09-378182 22438254

[32] Khan AR Hams E Floudas A Sparwasser T Weaver CT Fallon PG. PD-L1hi B cells are critical regulators of humoral immunity. Nat Commun (2015) 6:1–16. 10.1038/ncomms6997 25609381

[33] Velu V Titanji K Zhu B Husain S Pladevega A Lai L Enhancing SIV-specific immunity in vivo by PD-1 blockade. Nature (2009) 457:206–10. 10.1038/nature07662 19078956 PMC2753387

[34] Higuchi ML Brito T Reis MM Barbosa A Bellottii G Pereira-Barreto AC Correlation between *Trypanosoma cruzi* parasitism and myocardial inflammatory infiltrate in human chronic chagasic myocarditis: light microscopy and immunohistochemical findings. Cardiovasc Pathol (1993) 2:101–6. 10.1016/1054-8807(93)90021-S 25990604

[35] Sousa GR Gomes JAS Fares RCG Damásio MP Chaves AT Ferreira KS Plasma cytokine expression is associated with cardiac morbidity in Chagas disease. PLoS One (2014) 9:e87082–9. 10.1371/journal.pone.0087082 24603474 PMC3945957

[36] Pereira IR Vilar-Pereira G Moreira OC Ramos IP Gibaldi D Britto C Pentoxifylline reverses chronic experimental chagasic cardiomyopathy in association with repositioning of abnormal CD8+ T-cell response. PLoS Negl Trop Dis (2015) 9:e0003659. 10.1371/journal.pntd.0003659 25789471 PMC4366205

[37] Grabie N Gotsman I DaCosta R Pang H Stavrakis G Butte MJ Endothelial programmed death-1 ligand 1 (PD-L1) regulates CD8+ T-cell mediated injury in the heart. Circulation (2007) 116:2062–71. 10.1161/CIRCULATIONAHA.107.709360 17938288

