# Supplementary material for: Programmed Cell Death Protein 1–PDL1 Interaction Prevents Heart Damage in Chronic Trypanosoma cruzi Infection
Source: Front Immunol. 2018 May 7;9:997. doi: 10.3389/fimmu.2018.00997 (PMC5949529; doi:10.3389/fimmu.2018.00997)
Supplement: Supplementary file 1 [file Data_Sheet_1.docx]

Supplementary Material

PD1/PDL1 interaction prevents heart damage in chronic Chagas’ Disease

**Raíssa Fonseca^1*^, Rafael Moysés Salgado^1^, Henrique Borges da Silva^2^, Rogério Silva do Nascimento^1^, Maria Regina D’Império-Lima^1^, José Maria Alvarez^1*^**

*** Correspondence:** José Maria Álvarez Mosig: [jmamosig@icb.usp.br](mailto:jmamosig@icb.usp.br); Raíssa Fonseca**:** [raissaf@gmail.com](mailto:raissaf@gmail.com)

# Supplementary Figures

**Supplementary 1. Systemic parasitemia and histopathology score of mice treated with αPDL1 or and αPD1 associated with αPDL1 blocking antibodies.** C3H/HePAS mice were infected with 1x10^6^ *T. cruzi* parasites. At 330 dpi, mice were treated with 5 doses every 3 days (250 µg each) of anti-PDL1 (αPDL1) or anti-PD1 and anti-PDL1 (αPD1/αPDL1) blocking antibodies. Another group received only control IgG antibody (IgG) as a control. **(A)** Percentage of *T. cruzi*-positive LIT cultures containing 5 μL of blood samples before and after αPDL1 treatment. **(B)** Histopathology scores attributed to intensity of heart leukocyte infiltration in αPDL1 treated mice. **(C)** Percentage of *T. cruzi*-positive LIT cultures containing 5 μL of blood samples before and after αPD1 and αPDL1 treatment. **(B)** Histopathology scores attributed to intensity of heart leukocyte infiltration in αPD1 and αPDL1 treated mice. Statistical differences were evaluated between groups and indicated on graphs with * (p<0.05) or ** (p<0.01). Data are representative of one out of two independent experiments (n=13-14 each) showing mean ± sd.

**Supplementary 2. Early effects in circulating CD4^+^ and CD8^+^ T cell percentages after two doses of anti-PD1 and anti-PDL1 blocking antibodies associated with irradiated parasites.** Blood CD4^+^ and CD8^+^ T cell percentages before treatment (white boxes) and after the second dose of treatment (grey boxes) for IgG control treated chronic mice in comparison to mice that received anti-PD1, anti-PDL1 and irradiated *T. cruzi*. Data are grouped from three independent experiments (n=3 each). Box plots indicate medians (center lines), 25th and 75th percentiles (bottom and top box edges, respectively), minima and maxima (whiskers), and individual data points (circles).

**Supplementary 3. PBMC CD4^+^ and CD8^+^ T cells did not change CD69 or CD103 expression following treatment with anti-PD1 and anti-PDL1 blocking antibodies and irradiated parasites.** Percentage of PBMC CD4^+^ and CD8^+^ T cells expressing CD69 or CD103 after treatment. Data are grouped from three independent experiments (n=3 each). Box plots indicate medians (center lines), 25th and 75th percentiles (bottom and top box edges, respectively), minima and maxima (whiskers), and individual data points (circles).


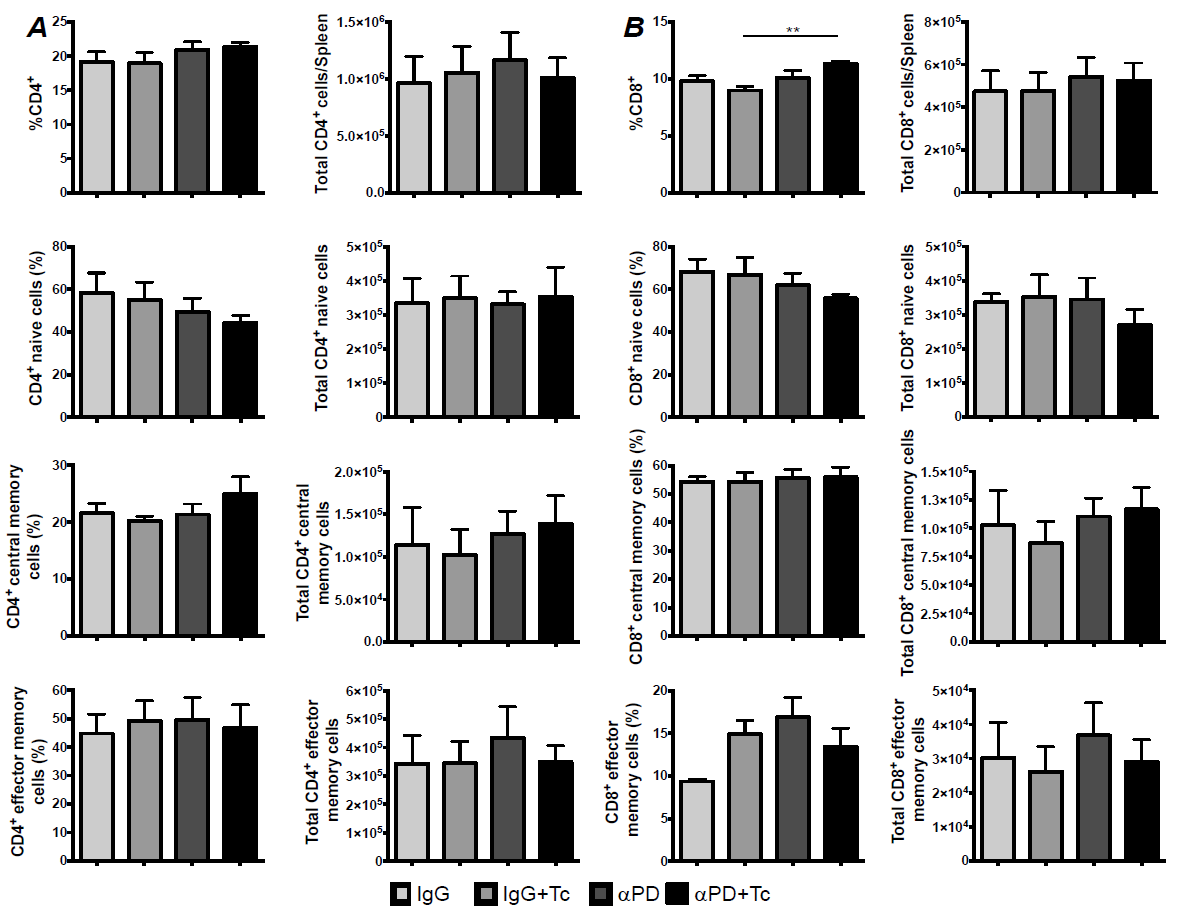


**Supplementary 4. Spleen CD4^+^ and CD8^+^ T cells did not change their phenotype upon treatment.** Percentage and total number of **(A)** CD4^+^ and **(B)** CD8^+^ T cells with naive, central memory or effector memory phenotype. Statistical differences were evaluated between groups and indicated on graphs with ** (p<0.01). Data are representative of one out of four independent experiments (n=3 each) showing mean ± sd.


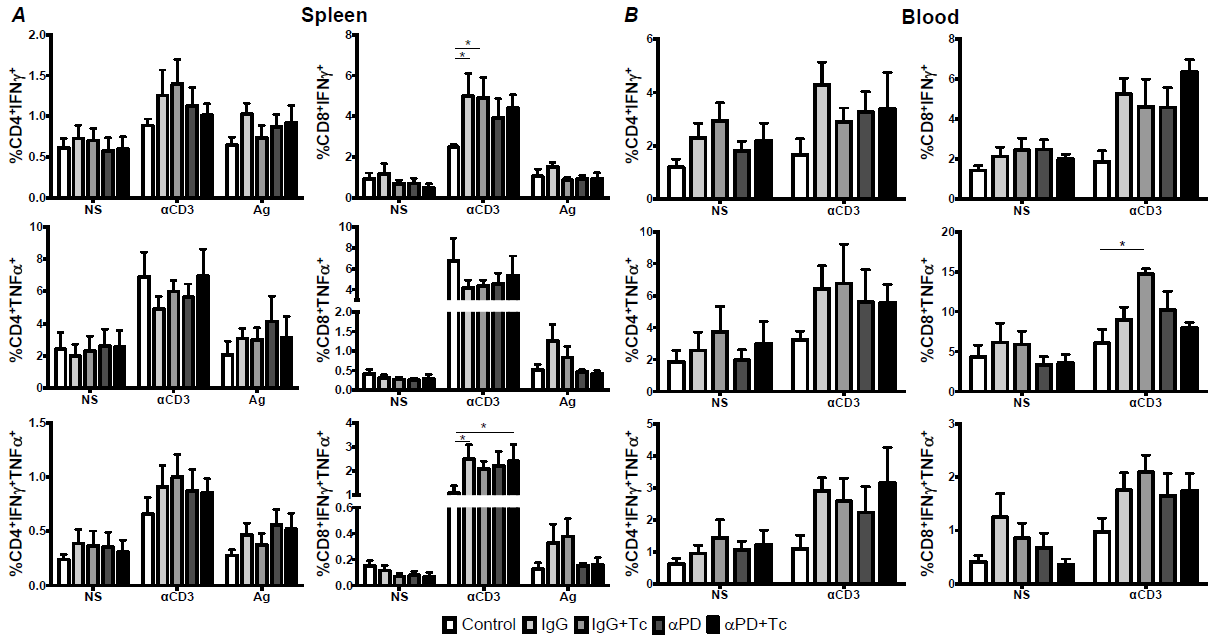


**Supplementary 5. *In vitro* IFNγ and TNFα production was not changed by *in vivo* treatment of spleen or blood CD4^+^ and CD8^+^ T cells.** Percentage of IFNγ and/or TNFα producing cells with αCD3 or *T. cruzi* antigen (Ag) after 12 hours in culture. **(A)** CD4^+^ and CD8^+^ splenocytes **(B)** CD4^+^ and CD8^+^ peripheral blood circulating cells. Statistical differences were evaluated between groups and indicated on graphs with * (p<0.05). Data are representative of one out of four independent experiments (n=3 each) showing mean ± sd.


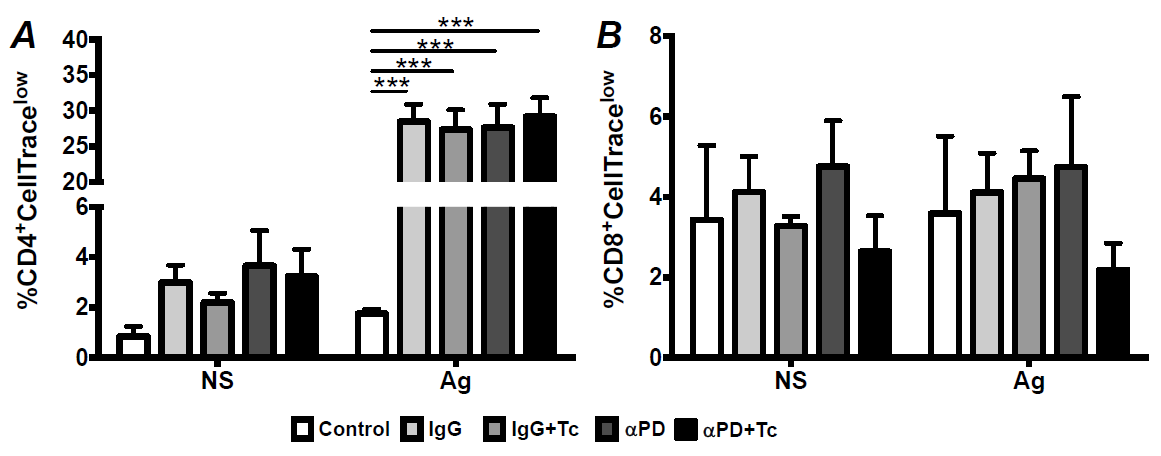


**Supplementary 6. *In vitro* proliferation of CD4^+^ and CD8^+^ splenocytes from treated mice is not enhanced by *in vivo* treatment. (A)** Percentage of CD4^+^CellTrace^low^ and **(B)** CD8^+^CellTrace^low^ kept for 72 hours without stimuli (NS) or with *T. cruzi* antigen (Ag) stimulation. Statistical differences were evaluated between groups and indicated on graphs with *** (p<0.001). Data are representative of one out of four independent experiments (n=3 each) showing mean ± sd.
